# Functional Analysis of *Plasmodium vivax Dihydrofolate Reductase-Thymidylate Synthase* Genes through Stable Transformation of *Plasmodium falciparum*

**DOI:** 10.1371/journal.pone.0040416

**Published:** 2012-07-09

**Authors:** Alyson M. Auliff, Bharath Balu, Nanhua Chen, Michael T. O’Neil, Qin Cheng, John H. Adams

**Affiliations:** 1 Drug Resistance and Diagnostics Department, Australian Army Malaria Institute, Enoggera, Queensland, Australia; 2 School of Population Health, University of Queensland, Brisbane, Queensland, Australia; 3 Department of Global Health, University of South Florida, Tampa, Florida, United States of America; 4 Division of Experimental Therapeutics, Walter Reed Army Institute of Research, Silver Spring, Maryland, United States of America; Université Pierre et Marie Curie, France

## Abstract

Mechanisms of drug resistance in *Plasmodium vivax* have been difficult to study partially because of the difficulties in culturing the parasite *in vitro*. This hampers monitoring drug resistance and research to develop or evaluate new drugs. There is an urgent need for a novel method to study mechanisms of *P. vivax* drug resistance. In this paper we report the development and application of the first *Plasmodium falciparum* expression system to stably express *P. vivax dhfr-ts* alleles. We used the *piggyBac* transposition system for the rapid integration of wild-type, single mutant (117N) and quadruple mutant (57L/58R/61M/117T) *pvdhfr-ts* alleles into the *P. falciparum* genome. The majority (81%) of the integrations occurred in non-coding regions of the genome; however, the levels of *pvdhfr* transcription driven by the *P. falciparum dhfr* promoter were not different between integrants of non-coding and coding regions. The integrated quadruple *pvdhfr* mutant allele was much less susceptible to antifolates than the wild-type and single mutant *pvdhfr* alleles. The resistance phenotype was stable without drug pressure. All the integrated clones were susceptible to the novel antifolate JPC-2067. Therefore, the *piggyBac* expression system provides a novel and important tool to investigate drug resistance mechanisms and gene functions in *P. vivax*.

## Introduction

*Plasmodium vivax* is the most widely distributed of the five known human malaria causing parasites and accounts for 80–391 million cases of malaria annually [1], [2], [3]. *P. vivax* infections are responsible for considerable morbidity and economic loss in endemic countries and have previously been thought to be rarely lethal. However, recent reports revealed that severe complications and death caused by *P. vivax* are not uncommon [2], [4], [5], [6]. Appropriate and timely treatment is the key to prevent morbidity and severe complications.

*P. vivax* parasites are susceptible to most antimalarials, particularly to chloroquine (CQ), which is used as the first line treatment for *P. vivax* infections in most areas of the world today. However, over the last twenty years there have been many reports that highlight the significant increase of resistance of *P. vivax* to CQ [7], [8], [9], [10], [11], [12], [13], [14], [15], [16], [17], [18], [19], [20] and to the sulfa/antifolate combination sulfadoxine/pyrimethamine (SP) [7], [21], [22], [23], [24], [25]. The spread of drug resistance in *P. vivax* makes the control and elimination of this species much more difficult.

A better understanding of mechanisms of drug resistance in *P. vivax* will facilitate the development of molecular methods for monitoring the spread and extent of resistance, which will guide national treatment policy and help the development of new drugs. However, investigations into genetic mechanisms of resistance in *P. vivax* have been severely restricted, due largely to difficulties to culture this species of parasites *in vitro*. Innovative methods are urgently needed to study drug resistance mechanism in *P. vivax*.

The genetic basis of *P. vivax* resistance to antifolates has been determined to be polymorphisms within the parasite’s dihydrofolate reductase (DHFR), a key enzyme in the folate biosynthetic pathway that is targeted by antifolates [26], [27], [28], [29], [30]. A particular set of mutations (encoding F57L + S58R+ T61M + S117T) within the *P. vivax dhfr* gene (*pvdhfr*) was shown to correlate with SP treatment failures [30], [31]. The role of mutant *pvdhfr* alleles to antifolate resistance was initially demonstrated in various heterologous expression systems, such as *E. coli* [32] and yeast [33], [34] because of the difficulties in culturing *P. vivax.*

More recently, we used a homologous *P. falciparum* episomal (transient) expression system [35], [36] to directly assess the effect of wild-type and various mutant *pvdhfr* alleles to parasites’ drug susceptibility. The study demonstrated that this episomal expression system has the potential to provide a rapid screening system for drug development and for studying the mechanism of resistance. However, transient *P. falciparum* expression systems tend to have copy number variability between parasite generations and rely on a constant drug selection pressure to ensure that the episomes are not lost over time. This constant drug pressure may also lead to other unforeseen effects on the parasite.

In recent years a *piggyBac* mediated genome integration method has been developed enabling stable transgene expression in *P. falciparum* [37], [38], [39]. The *piggyBac* transposition system for *P. falciparum* involves a transiently expressed helper plasmid (transposase) that is used to activate the insertion of the transposon plasmid. This transposon contains the positive selectable marker and the gene of interest expression cassette flanked by the Inverted Terminal Repeat (ITR) sequences. Upon expression in the parasite, the *piggyBac* transposase identifies a TTAA target in the *P. falciparum* genome, cuts into this position within the genome and then inserts the expression cassette from within the transposon into the *P. falciparum* genome. Due to the orientation of the ITRs in the transposon only the expression cassette is integrated and the remainder of the plasmid is left outside [40], [41]. The *piggyBac* transposition system has been shown previously [41], [42] to rapidly and efficiently create stable genomic integrations within a few weeks as opposed to 6–12 months compared to homologous recombination.

To date, there are limited reports of the stable transfection of *P. vivax* genes into the *P. falciparum* genome [43], [44], [45]. In this article we report the development of the *piggyBac* transposition system for the integration of *pvdhfr* into the *P. falciparum* genome, the transcription of the *pvdhfr* gene, and the response of the *pvdhfr* alleles to conventional antifolate drugs as a proof of concept for the utility of the system. We have also compared the phenotype stability between *piggyBac pvdhfr* integrants and the transiently expressed *pvdhfr* [35]. To demonstrate the usefulness of the expression system for screening and evaluating new drugs against *P. vivax* we also used this expression system to test the inhibitory effect of a new generation antifolate, JPC-2067, which is an active dihydrotriazine metabolite of JPC-2056. JPC-2056 is a third generation phenoxypropoxybiguanide prodrug that was synthesized [46] to overcome the poor gastrointestinal tolerability associated with WR99210, as well as the safety and regulatory restrictions associated with the WR99210 precursor PS-15 [47], [48]. The JPC-2067 metabolite has been found to be as potent as WR99210 *in vitro* against *P. falciparum* pyrimethamine sensitive and resistance strains and has also been selected as the lead candidate for pre-clinical development based on the equivalent efficacy to PS-15 and comparable oral tolerability in mice and monkeys to proguanil [46], [49]. The findings will help the further development of this compound.

## Results

### Transfection and Insertion Site Analysis

Three alleles of the *pvdhfr* gene, wild-type, single mutant (117N) and quadruple mutant (57L, 58R, 61M and 117T) alleles were cloned into the *piggyBac* transposition vector and transfected into the *P. falciparum* line NF54. The transfected parasites were selected under blasticidin selection pressure. Blasticidin resistant populations were selected rapidly within 2–3 weeks and the total number of *piggyBac* insertions obtained per transfected population varied between 1 and 3. Through 6 independent transfections, blasticidin selection pressure and limiting dilution procedures, 16 unique clones of *P. falciparum* with *piggyBac* insertions in their genomes were generated. Southern blot hybridization analysis performed on these clones, revealed that a single *piggyBac* insertion occurred in each clone (Table 1) and that none of the clones retained the *piggyBac* plasmid as episomes indicating highly efficient transposition events (data not shown). Importantly, none of the transfected *P. falciparum* clones displayed any growth defects as judged by parasite morphology and development.

**Table 1:** *pvdhfr* alleles inserted and insertion sites in the integrated clones.

Plasmid Name	*Pvdhfr* Amino Acid Mutations							Clone	Insertion Site		
	33	50	57	58	61	117	173		Inter/intragenic	Genes	Chromosome
TruncatedPv bsd								B8	Intergenic	GTP binding protein - tRNA binding protein	14
								G3	Intergenic	MAL8P1.156 - MAL8P1.154	8
*pvdhfr*wild-type allele	P	N	F	S	T	S	I	I	Intragenic	PFD0885	4
								K	Intragenic	PFL0190w	9
								D4	Intergenic	PF10_0114 - PF10_0015	10
*pvdhfr* single mutant allele	P	N	F	S	T	**N**	I	E	Intergenic	PF11_0047 - PF11_0048	11
								H7	Intergenic	PF10_0182 - PF10_0183	10
								A4	Intergenic	PFF0825c - PFF0830w	6
								C5	Intergenic	PF14_0729 - etramp14.2	14
								B7	Intergenic	PF13_0061 - PF13_0062	13
								D12	Intergenic	PF11_0151 - PF11_0153	11
								E1	Intergenic	PF14_0071 - PF14_0072	14
*pvdhfr* quadruple mutant allele	P	N	**L**	**R**	**M**	**T**	I	C3	Intragenic	813289	9
								B3	Intergenic	MAL13P1.114 - MAL13P1.115	13
								D1	Intergenic	pHISTa-like - plasmodium exported protein(hyp12)	13
								H8	Intergenic	phosphomannomutase putative - a conserved plasmodium protein	10

All of the *piggyBac* insertions could be mapped unambiguously on the *P. falciparum* genome by performing BLAST searches using the NCBI http://www.ncbi.nlm.nih.gov/genome/seq/BlastGen/BlastGen.cgi?taxid=5833 database. The sites of integration in the transformed populations were identified by either Thermal asymmetric interlaced (TAIL) PCR or vectorette PCR. The vectorette PCR was more superior to TAIL PCR in determining the integration sites in terms of sensitivity and specificity (data not shown). All *piggyBac* insertions identified were at the expected TTAA target sequence sites and their insertion sites distributed throughout the *P. falciparum* genome with no bias for any particular chromosome. Of the 16 *piggyBac* insertions 13 (81.25%) were identified in intergenic regions while the 3 remaining *piggyBac* insertions occurred within coding regions. Table 1 summarizes the *pvdhfr* allele inserted and insertion sites of these clones.

### Transcription and Expression of the pvdhfr Wild-type and Mutant Alleles in P. falciparum Erythrocyte Stage Parasites

To demonstrate the transcription and expression levels of the integrated *pvdhfr* gene, we selected a minimum of two clones from each *pvdhfr* allele that have different insertion sites to firstly determine the transcription of the endogenous *pfdhfr*, and secondly determine the transcription and expression of the *pvdhfr* alleles in these clones by conducting both real-time RT-PCR and Western blots.

### 1) Transcription of Endogenous pfdhfr Over the Erythrocytic Life Cycle

**Figure 1:**
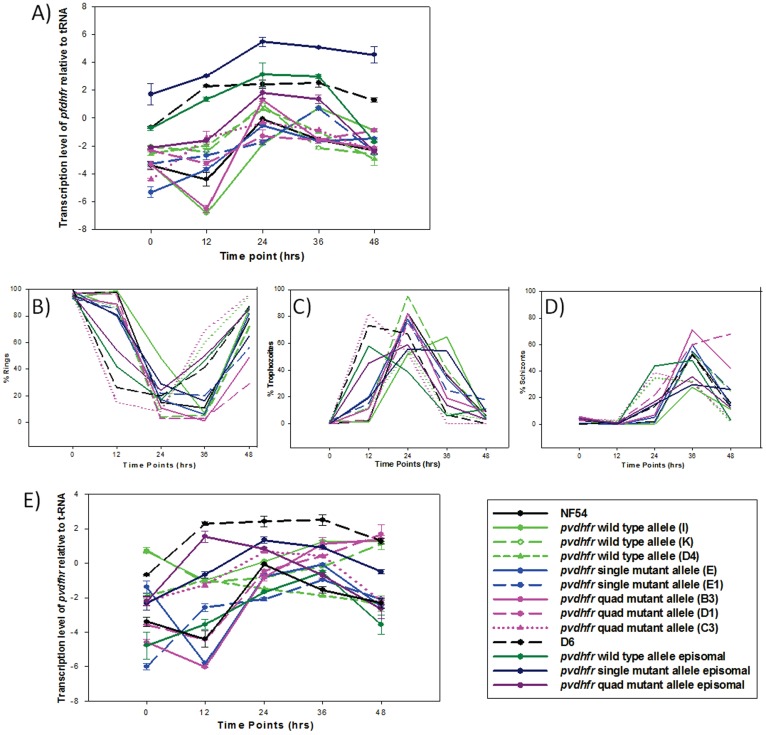
Transcription profile and parasite stages of *dhfr* mRNA expression over the parasite life cycle. A) Transcription levels of the *pfdhfr* relative to that of *seryl-tRNA* gene. B) % rings over the parasite life cycle. C) % trophozoites over the parasite life cycle. D) % schizonts over the parasite life cycle. E) Transcription levels of *pvdhfr* relative to that of *seryl-tRNA*. Note: NF54 and D6 in panel E represent level of *pfdhfr* transcription as a comparison.

The transcription levels of the *pfdfhr* gene relative to that of the seryl-tRNA synthetase (seryl-tRNA) gene (PR07-0073) was determined every 12 hrs, starting with rings (time 0 hrs), through trophozoites (12–24 hrs), schizonts (24–36 hrs) and ending at rings (48 hrs) (Figure 1A). The proportions of ring, trophozoite and schizont at each time point are shown in Figure 1B, C and D. In the parental NF54 line, the transcription level of *pfdhfr* increased as the parasite developed from ring stage to trophozoite stage and peaked at late trophozoite stage (at 24 hrs). All integrated clones showed a similar *pfdhfr* transcription profiles as the parental NF54 strain except clone I (expressing a wild-type *pvdhfr*) and clone E1 (expressing a single mutant), which had a transcription peak at 36 hrs. The overall transcription levels of *pfdhfr* in all integrated clones were lower than that of the seryl-tRNA (Figure 1A). In contrast, the overall transcription levels of *pfdhfr* in D6 and episomally transfected D6 lines (containing *pvdhfr* as episomes) were generally higher than or comparable to that of seryl-tRNA (Figure 1A).

### 2) Transcription of Transfected pvdhfr Over the Erythocytic Life Cycle

To determine if the transcription and expression of *pvdhfr* driven by a *P. falciparum dhfr* promoter coincides with the expression of the endogenous *pfdhfr*, the *pvdhfr* transcription profile was compared to that of *pfdhfr* within one erythrocytic cycle. The *pvdhfr* transcription profiles of the integrated clones were less homogenous compared to the transcription of the endogenous *pfdhfr* peaking between 24 and 48 hrs, with the overall level of transcription lower than that of seryl-tRNA (Figure 1E). Episomally expressed *pvdhfr* alleles also peaked at trophozoite and schizont stages (Figure 1E). A one way ANOVA analysis showed that there was no statistically significant difference (P = 0.123) in *pvdhfr* transcription level between the differently transfected parasites.

### 3) Transcription of pvdhfr in Different Clones

To determine if the location of the transgene integration affects expression of the parasites *pvdhfr,* we compared clones that had integrated within the coding region to those integrants that had integrated into non-coding regions. No statistical significance (P = 0.432) of the *pvdhfr* transcription was found.

### 4) Protein Expression of Transfected pvdhfr Gene

The protein expression was investigated using both anti-*cmyc* and anti-*bsd* antibodies. Neither antibody produced a positive signal on Western blot, while a Rex-1 antibody (kindly supplied by Dr Don Gardiner, QIMR) recognized parasite proteins on the same blot (data not shown).

### Susceptibility to Conventional Antifolates

**Figure 2:**
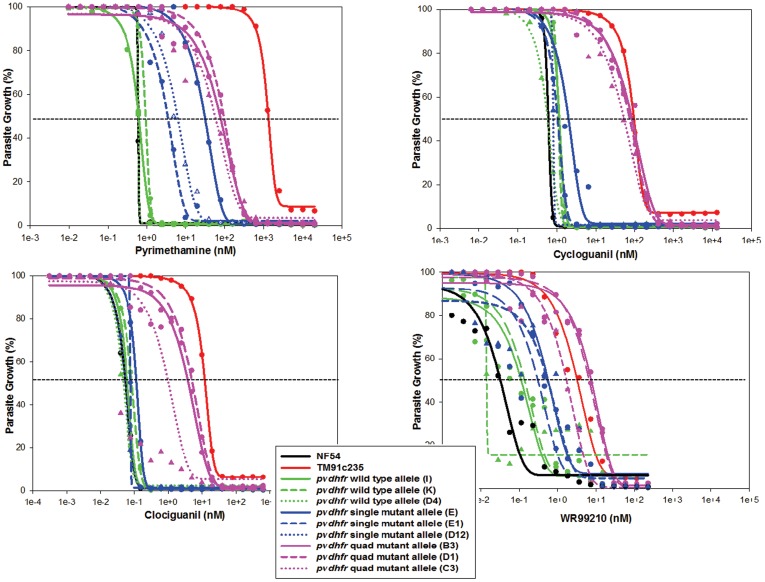
*In vitro* susceptibility of *P. falciparum* NF54 and integrated parasite clones stably expressing various *pvdhfr* mutants to DHFR inhibitors: pyrimethamine (top left panel), cycloguanil (top right panel), clociguanil (bottom left panel), and WR99210 (bottom right panel). Each color represents a *pvdhfr* allele and lines of the same color represent different clones of the same expressed *pvdhfr* allele. The symbols represent the means of triplicate data points.

Three different clones of each *pvdhfr* allele transfectants were assayed using the *in vitro* susceptibility assay to determine firstly, if the susceptibility profile was the same between the different clones with the same *pvdhfr* allele and secondly, to assess the direct effect the different *pvdhfr* alleles had on the parasites susceptibility to pyrimethamine, cycloguanil, clociguanil and WR99210 (Table 2 and Figure 2). The three clones transfected with wild-type *pvdhfr* exhibited similar susceptibility to all drugs (P>0.05). The three clones expressing single mutant *pvdhfr* allele showed comparable susceptibility to cycloguanil, clociguanil and WR99210 with a maximum 2.4, 2.2 and 1.8 fold difference in IC_50_ values between the clones (P>0.05) for cycloguanil, clociguanil and WR99210, respectively. However, one single mutant clone (E) exhibited a 10 fold higher IC_50_ value to pyrimethamine compared to two other clones expressing the same allele. The *pvdhfr* quadruple mutant clones, B3 and D1 had a similar susceptibility profiles for all the antifolates (P>0.05), however, clone C3 was significantly different compared to the other two quadruple mutant clones (P<0.05).

**Table 2:** Susceptibility of parasites and transfected parasite lines to pyrimethamine, cycloguanil, clociguanil, WR99210 and JPC-2067.

Parasite/Transfected Clone	Pyrimethamine		Cycloguanil		Clociguanil		WR99210		JPC-2067	
	IC_50_ (± SD)	RR	IC_50_ (± SD)	RR	IC_50_ (± SD)	RR	IC_50_ (± SD)	RR	IC_50_ (± SD)	RR
**Native Parasite Strain**										
**NF54**	0.61 (±0.00)	1	0.61 (±0.09)	1	0.05 (±0.02)	1	0.03 (±0.00)	1	0.05 (±0.00)	1
**TM91c235**	1276.60 (±2.32)	2103	85.58 (±15.20)	74	11.19 (±2.10)	211	5.70 (±0.26)	190	0.06 (±0.26)	1
**Clones of NF54 integrated with different ** ***pvdhfr*** ** alleles**										
**Wild-type (I)**	0.56 (±0.18)	1	1.16 (±0.29)	2	0.06 (±0.02)	1	0.10 (±0.01)	4	0.14 (±0.01)	3
**Wild-type (K)**	0.94 (±0.01)	2	1.10 (±0.12)	2	0.08 (±0.06)	2	0.16 (±0.00)	5	0.12 (±0.00)	3
**Wild-type (D4)**	0.61 (±0.01)	1	0.57 (±0.12)	1	0.05 (±0.02)	1	0.01 (±0.02)	0	0.09 (±0.02)	2
**Single mutant (E)**	30.20 (±7.32)	50	1.92 (±0.37)	3	0.11 (±0.04)	2	0.53 (±0.09)	18	0.12 (±0.09)	3
**Single mutant (E1)**	3.31 (±1.51)	5	1.03 (±0.11)	2	0.08 (±0.01)	1	0.32 (±0.25)	11	0.07 (±0.25)	2
**Single mutant (D12)**	5.85 (±0.09)	10	0.81 (±0.02)	1	0.05 (±0.02)	1	0.57 (±0.27)	19	0.11 (±0.27)	2
**Quadruple mutant (B3)**	75.06 (±37.00)	134	87.51 (±24.67)	144	4.04 (±1.92)	76	7.23 (±0.09)	241	0.03 (±0.02)	1
**Quadruple mutant (D10)**	93.02 (±30.05)	166	73.21 (±20.23)	121	5.14 (±1.86)	97	6.68 (±0.38)	223	0.06 (±0.04)	1
**Quadruple mutant (C3)**	57.11 (±16.97)	94	119.00 (±3.90)	196	1.02 (±0.10)	19	1.57 (±0.01)	52	0.05 (±0.01)	1
**Clones of D6 episomally expressing different ** ***pvdhfr*** ** alleles**										
**Wild-type**									0.17 (±0.01)	4
**Single mutant**									0.19 (±0.02)	4
**Quadruple mutant**									1.03 (±0.30)	22

IC_50_s are means of triplicate data points and are expressed as mean nM ± SD. The relative resistance index (RR) was determined in comparison to NF54.

The clones of NF54 transfected with the wild-type *pvdhfr-ts* (WT) had a similar susceptibility profile to that of the parental NF54 strain. This demonstrated that the WT *pvdhfr* allele was equally susceptible to antifolates compared to the wild-type *pfdhfr-ts* (P = 0.477). In contrast, the NF54 transfected with the mutant *pvdhfr* alleles were less susceptible to antifolates compared to the WT. NF54 clones transfected with the single mutant *pvdhfr* 117N were 5 to 50 fold less susceptible to pyrimethamine (P = <0.001) and 11 to 19 fold less susceptible to WR99210 (P = 0.013) compared to the clones with wild-type *pvdhfr* allele. However, their susceptibilities to cycloguanil and clociguanil were not different to the clones with the wild-type *pvdhfr* allele (P = 0.469 and P = 0.440).

The *pvdhfr* quadruple mutants (57L/58R/61M/117T) that were integrated into NF54 parasites were relatively more resistant to all the antifolates tested than the WT and single mutant *pvdhfr* 117N allele transfected clones. The quadruple mutant *pvdhfr* allele transfectants were 94–166, 121–196, 19–97 and 52–241 fold more resistant to pyrimethamine, cycloguanil, clociguanil and WR99210 respectively, compared to the NF54 parental line (P = <0.005), but were 13–22 and 2–11 fold less resistant to pyrimethamine and clociguanil, respectively, than the native *P. falciparum* quadruple mutant, TM91c235 (P = <0.001 and P = 0.003, respectively) (Table 2). Each clone was assayed three times on three different occasions and the results were comparable in terms of the rank order and differences between alleles (data not shown).

To confirm that the above described changes in susceptibility to antifolate drugs were not due to mutations or amplification that had occurred in the endogenous *pfdhfr* in response to the selection pressure with blasticidin, we sequenced the *pfdhfr* and measured the copy number of *pfdhfr* for all the transfected clones. No mutations within the open reading frame of *pfdhfr* and no copy number changes were identified in the integrated parasites (data not shown). Furthermore, to determine if any plasmid components other than *pvdhfr* had any effect on the susceptibility to antifolate drugs, we compared the NF54 transfected with a truncated *bsd pvdhfr* to NF54 transfected with the *P. vivax* wild-type *dhfr-ts* (WT) and found no difference (data not shown).

### Susceptibility to a Novel Antifolate, JPC-2067

**Figure 3:**
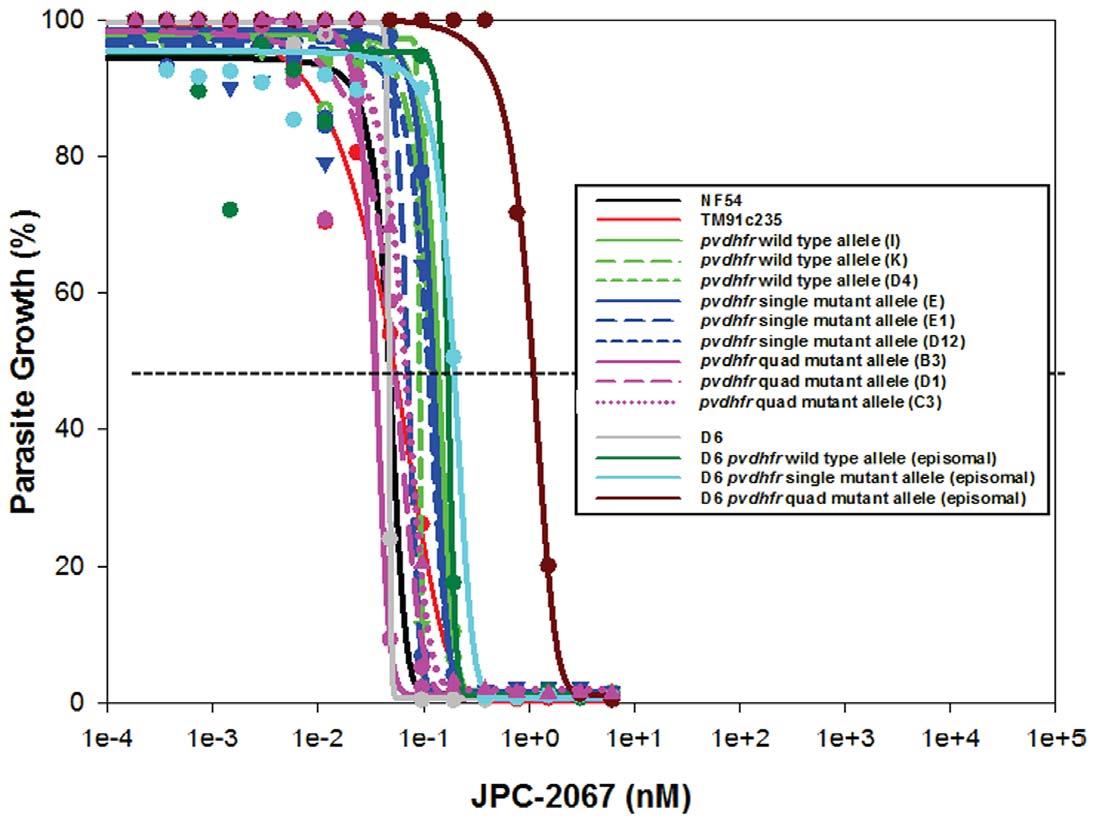
*In vitro* susceptibility profiles of *P. falciparum* NF54, NF54 stably transfected parasites, D6, D6 episomally transfected parasites and TM91c235 to JPC-2067. The symbols represent the means of triplicate data points.

The purpose of being able to express *P. vivax* drug resistance genes in *P. falciparum* is so that we can better understand the effect that the various mutant alleles will have against new compounds. Table 2 and Figure 3 show the effect of JPC-2067 against the wild-type and various mutant *pvdhfr* integrated alleles. The *P. falciparum* lines of NF54 (carrying wild-type *pfdhfr*) and TM91c235 (carrying quadruple mutant *pfdhfr*) were equally susceptible to JPC-2067(P = >0.05). All of the integrated clones with different *pvdhfr* alleles had a similar susceptibility profile as NF54 and TM91c235, with a maximum difference of 4 fold in IC_50_ values between them (P>0.05).

### Stability of Copy Number (CN) and the Susceptibility to Conventional Antifolates

To determine if the removal of drug selection pressure on the transfected parasites resulted in a change in CN and/or the susceptibility of transfected parasites to antifolates, six integrated clones (two wild-type, two single and two quadruple mutant clones) were monitored for 32–53 erythrocytic cycles (64–106 days) after selection pressure was withdrawn. Sample were taken at 4–5 time points and used to assess CN and susceptibility to antifolates. D6 parasites expressing various *pvdhfr* alleles as episomes [35], [36] were also monitored in parallel for CN and susceptibility with and without drug pressure.

For the six integrated NF54 clones, the CN of *pvdhfr* remained at approximately one throughout the 32 cycles with no significant changes in parasite susceptibility to pyrimethamine. For the episomal transfected lines, CN did not reduce with time when cultured under drug pressure. However, CN reduced over time in single and quadruple mutant transfectants when cultured without drug pressure. It was observed that approximately one episome was lost every 9 cycles for the single mutant and every 4 erythrocytic cycles for quadruple mutant transfected parasites on average (Table 3). As the CN of the quadruple mutant *pvdhfr* episomes decreased, the susceptibility of the parasite to pyrimethamine increased significantly (Spearman test, P = 0.0167). The same trend was seen in the single mutant although Spearman test was just above significance (P = 0.0833). No significant relationship was observed between the CN of the episomes and the susceptibility to pyrimethamine for the wild-type (P = 0.7707). This trend was also observed for cycloguanil, clociguanil and WR99210 (data not shown). It was also observed that there was a significant difference between the IC_50_ values of the integrated quadruple *pvdhfr* allele and the episomally expressed quadruple *pvdhfr* allele (P = 0.001).

**Table 3:** Copy number of *pvdhfr* (CN) and IC_50_ to pyrimethamine of transfected parasites at five time points over 53 parasite life cycles.

Parasite strain/transfected clones	Number of days since the start of experiment											
	0		24			46		64		106		
	CN	IC_50_ (± SD)	CN		IC_50_ (± SD)	CN	IC_50_ (± SD)	CN	IC_50_ (± SD)	CN	IC_50_ (± SD)	
**Native parasite strains**												
**D6**	0	**1.41 (±0.42)**	0	**0.95 (±0.32)**		0	**1.19 (±0.17)**	0	**1.19 (±0.52)**	0	**0.81 (±ND)**	
**NF54**	0	**0.78 (±0.05)**	0	**0.94 (±0.03)**		0	**0.74 (±0.15)**	0	**0.64 (±0.16)**	0	ND	
**TM91c235**	0	**2067.00 (±162.00)**	0	**2526.00 (±420.00)**		0	**2760.00 (±72.00)**	0	**1126.00 (±27.30)**	0	**2252.00** **(± ND)**	
**D6 parasites episomally expressing various ** ***pvdhfr*** ** alleles cultured without and with drug selection**												
**Wild-type**	1.65	**0.78 (±0.07)**	2.03	**0.54 (±0.26)**		2.59	**0.59 (±0.11)**	2.61	**0.59 (±0.16)**	2.39	**0.56 (±0.04)**	
**Wild-type + blasticidin pressure**	1.65	**0.78 (±0.07)**	2.17	**0.69 (±0.13)**		1.98	**0.73 (±0.12)**	1.92	**0.72 (±0.17)**	2.16	**0.35 (±0.01)**	
**Single mutant**	11.94	**1.57 (±0.50)**	8.10	**0.20 (±0.06)**		8.90	**0.87 (±0.07)**	7.00	**0.32 (±0.10)**	5.70	**0.13 (±0.02)**	
**Single mutant + blasticidin pressure**	11.94	**1.57 (±0.50)**	13.83	**0.45 (±0.09)**		16.95	**1.08 (±0.08)**	15.75	**0.99 (±0.13)**	19.00	**0.41 (±0.12)**	
**Quad mutant**	15.99	**1698.00 (±547.00)**	6.94	**725.00 (±128.00)**		4.71	**636 (±81.00)**	4.52	**507.00 (±63.00)**	1.24	**94.71 (±4.40)**	
**Quad mutant +pyr pressure**	15.99	**1698.00 (±547.00)**	11.09	**1179.00 (±74.00)**		10.42	**1393.00 (±224.00)**	11.35	**1156 (±186.00)**	33.00	**1011.18 (±110.00)**	
**NF54 parasite clones integrated with different ** ***pvdhfr*** ** alleles cultured without drug selection**												
**Wild-type (I)**	0.99	**0.93 (±0.39)**	1.18	**0.54 (±0.47)**		0.74	**2.82 (±0.93)**	1.20	**0.46± (0.93)**	ND		ND
**Wild-type (D4)**	0.63	**0.71 (±0.34)**	1.19	**0.56 (±0.24)**		1.19	**1.50 (±0.34)**	1.19	**0.53 (±0.07)**	ND		ND
**Single mutant (E)**	0.50	**79.40 (±8.66)**	0.80	**43.2 (±7.32)**		0.72	**65.58 (±3.84)**	ND	**42.3 (±6.35)**	ND		ND
**Single mutant (E1)**	0.60	**8.31 (±1.51)**	ND	**7.89 (±0.83)**		0.54	**4.10 (±0.16)**	ND	**1.41 (±0.30)**	ND		ND
**Quad mutant (B3)**	1.15	**68.42 (±6.78)**	0.70	**137.08 (±0.93)**		0.92	**84.93 (±6.78)**	0.83	**201 (±51.99)**	ND		ND
**Quad mutant (C3)**	0.84	**69.38 (±2.62)**	0.86	**53.64 (±2.62)**		1.20	**94.6 (±59.86)**	0.82	**183.00 (±22.13)**	ND		ND

## Discussion

This study demonstrates the integration and stable expression of *pvdhfr* alleles in a heterologous system of *P. falciparum*. It provides evidence that the transposon-mediated genomic integration method, *piggyBac*, is a useful method to create stable transformed clones for studying *P. vivax* drug resistance mechanisms and gene functions in *P. falciparum.* This new system will enable studies into the biology of *P. vivax* parasites which had been significantly lacking due, partially to, our inability to culture this pathogenic parasite species.

The *piggyBac* transfections inserted a single copy of *pvdhfr* into one of the *P. falciparum* chromosomes. The insertion sites appear to be random with the majority of sites identified in non-coding regions of different chromosomes as previously described [41], [42]. The insertion of *pvdhfr* did not affect the timing and level of endogenous *pfdhfr* as the transcription timing and levels of endogenous *pfdhfr* in all clones were comparable to those of the parental untransfected parasites. The location of the insertions, which were identified in this study, also did not appear to affect the transcription level of transfected *pvdhfr* because the transcription levels were not significantly different between clones that were inserted in different locations on different chromosomes. Interestingly, the peak transcription time of *pvdhfr* (∼36 hrs in the experiment) appears to lag behind that of the endogenous *pfdhfr* (∼24 hrs in the experiment). Since the promoter used to transcribe *pvdhfr* is the *pfdhfr* promoter, it is not clear why this shift of timing has occurred. Differences in developmental stages of parasites should not have resulted in this shift as the transcription of *pfdhfr* and *pvdhfr* were measured in the same sample. One possibility may be due to the epigenetic control at the *pfdhfr* locus.

The protein expression was also investigated using both anti-*cmyc* and anti-*bsd* antibodies. Unfortunately, neither antibody produced a positive signal on Western blot. There are several possible explanations for negative Western blot. Firstly, the amount of protein expressed was too small for these antibodies to bind and generate a positive signal. Earlier reports [50], [51], [52] also described failure to obtain positive signals on Western blot and demonstrated that this was due to DHFR-TS being a low abundance protein. Secondly, the expressed PvDHFR maybe conformationally folded such that the *cmyc* and *bsd* were not accessible to the antibodies. Nevertheless, the transfected parasites had a susceptibility profile corresponding to the transfected *pvdhfr* allele, indicating the successful expression of functional PvDHFR in the transfected *P. falciparum* parasites.

As was seen previously in the episomal expression of the *pvdhfr* alleles [35], [36], the same *in vitro* susceptibility phenotypic profile was observed for the integrated wild-type and mutant *pvdhfr* alleles. That is, the wild-type *pvdhfr* transfected clones were as susceptible to antifolates as the susceptible parent strain while the mutant *pvdhfr* alleles conferred significantly reduced susceptibility to the antifolate drugs; with the quadruple mutant *pvdhfr* allele conferring a higher resistance than the single mutant *pvdhfr* allele tested. This increase in resistance to the antifolates was not due to mutations in the endogenous *pfdhfr*, nor was it due to amplification of the endogenous *pfdhfr* gene or changes in its expression as all clones had a comparable timing and level of *pfdhfr* transcription.

Three different clones of the same *pvdhfr* allele (wild-type, single and quadruple mutant) were used to determine the susceptibility phenotype of the transfected parasites to rule out the possible effect of other unintended mutations or unintended positional effects caused by the integration of the transfected allele into the parasite genome [53]. Indeed, while consistent susceptibility phenotype profiles were observed between the three wild-type clones (I, K and D4), between two single mutant clones (D12 and E1) and between two quadruple mutant clones (B3 and D10), significantly different phenotypes were observed in one of the three single mutant clones (E) and one of the three quadruple mutant clones (C3). This highlights the importance of using multiple clones with different insertion sites to determine the phenotype profile of transfected parasites.

Interestingly, the level of resistance transferred by two systems was not identical, particularly in susceptibilities to antifolates between episomal and integrated quadruple mutant *pvdhfr* allele (57L/58R/61M/117T) transfected parasites. The integrated parasite clones showed a lower level of resistance to all antifolate drugs compared to episomal transfected parasites. The IC_50_ values to pyrimethamine was 75.06±37.00 nM, 57.11±16.97 nM and 93.02±30.05 nM for integrated clones and 1698±547 nM for the episomal transfected parasite line. The quadruple mutant *pvdhfr* integrated clones also showed a lower level of resistance to the new antifolate compound JPC-2067 (resistance index 0.72–1.26) than the episomal transfected parasites (resistance index 22) [49]. The difference in susceptibility to these drugs is likely resulted from difference in *pvdhfr* copy number between the episomal and integrated quadruple mutant parasites. When the copy number of *pvdhfr* episomes reduced to 1.24 after culturing 106 days without drug selection, the IC_50_ value of the episomal quadruple mutant parasite decreased to 94.71±4.4 nM, comparable to that of integrated quadruple mutant parasite.

The relationship between copy number and susceptibility to antifolate drugs has been studied in the past without a clear conclusion. Results of our previous study [35], suggested that the susceptibility of the antifolates was not affected by the copy number of the episomes carrying the wild-type and mutant *pvdhfr* alleles. In the current study, we conducted a test over 106 days on the same parasite line and identified a good correlation over time between episome copy number and IC_50_ values to pyrimethamine for both the single and quadruple mutant *pvdhfr* transfected parasites, while no such a correlation was identified for the wild-type *pvdhfr* transfected parasites. The results demonstrate that variation in copy number within the same transfectant parasite line will cause changes in parasite susceptibility to antifolates.

The development of a homologous system to stably express *P. vivax* genes removes the variability and uncertainty in parasite susceptibility caused by episomal copy number and allows us to determine more accurately the susceptibility of antifolates against parasites with mutant *pvdhfr* alleles. This system has a better potential to provide a rapid screening system for drug development and for studying drug resistance mechanisms in *P. vivax* than the episomal expression system. To demonstrate this potential we tested a novel antifolate compound JPC-2067 against parasite clones transfected with various *pvdhfr* alleles. This compound when given to monkeys as a pro-drug (JPC-2056) has been shown to be highly potent against *P. falciparum* parasite carrying wild-type and mutant *pfdhfr* alleles [49]. However, its effect against *P. vivax* carrying different *pvdhfr* was unknown causing concerns on the further development of this compound. Using the stably transfected parasites we demonstrated that the compound was highly effective in killing parasites integrated with *pvdhfr* quadruple mutant allele with the same potency as against parasites integrated with wild-type *pvdhfr.* These results provide evidence and support for the future development of JPC-2056 for the treatment of both *P. falciparum* and *P. vivax* infections. In addition to assisting drug development, this stable *P. falciparum* expression system has the potential to identify and confirm other *P. vivax* drug targets, as well as elucidate the biological function of other *P. vivax* genes.

## Materials and Methods

### Parasites

*P. falciparum* lines NF54 and TM91c235 were used in this study as antifolate susceptible and resistant controls, respectively. Parasite lines were cultured continuously in a 4% hematocrit blood-LPLF RPMI 1640 as previously reported [54]. Parasite cultures were synchronized at the ring stage by repeated D-Sorbitol treatment [55] and were allowed to grow for one cycle after the last treatment and then used for transfection or *in vitro* susceptibility testing. *P. falciparum* D6 and D6 episomally expressing various alleles of *pvdhfr* [35] were used in copy number, transcription and susceptibility tests. For CN and transcription analyses, aliquots of parasite cultures were taken every 12 hrs, starting from early ring stage, over a period of 48 hrs.

### Plasmid Construction

**Figure 4:**
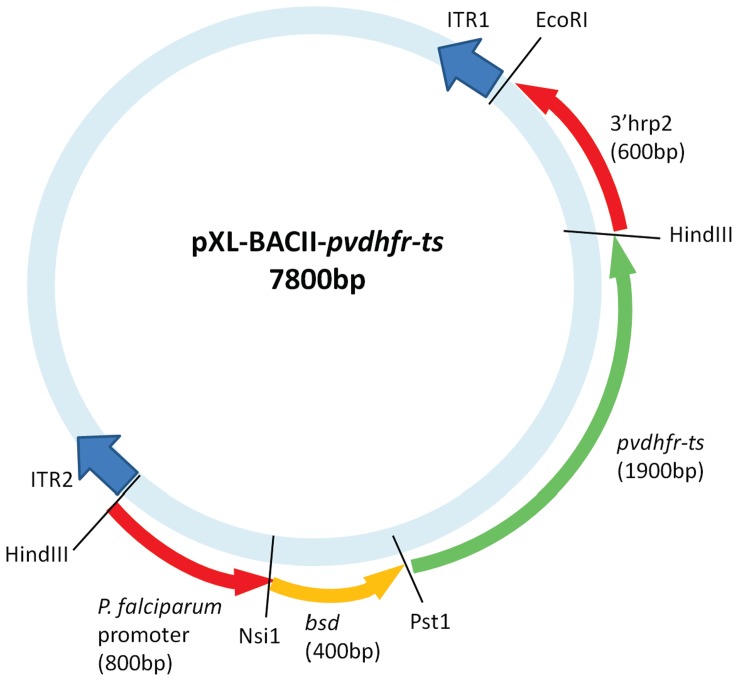
pXL-BACII-*pvdhfr* plasmid design for *piggyBac* transformation of *P. falciparum*. The pXL-BacII-DHFR vector [42] was cut with *Hind*III to remove the 5′ *calmodulin* and *hdhfr* fragments from the pXL-BacII vector. The *pfdhfr promoter-bsd-cmyc-pvdhfr-ts* cassette was excised from the pRSET-C vector as a 3.1 kb *Hind*III fragment and cloned into pXL-BacII, such that it is flanked by the *piggyBac* ITR2 and 3′ *histidine-rich protein-2* (*hrp2*). The sizes of the boxes are not proportional to the lengths of the genes. The *pfdhfr* promoter drives the expression of *bsd-cmyc-pvdhfr-ts* fused proteins.

The open reading frames (ORFs) of wild-type, single mutant (117N) and quadruple (57L/58R/61M/117T) mutant alleles of *pvdhfr-ts* were amplified from genomic DNA of *P. vivax* isolates [56] by PCR using primers and conditions described previously [30], [56]. The plasmid construction was performed as previously described [35], [36] with minor modifications. The *pvdhfr* PCR product was inserted between the *P. falciparum dhfr-ts* promoter and *P. vivax thymidylate synthase (pvts);* the *pvdhfr* cassette had the 0.4-kb *Saccharomyces cereviseae bsd* ORF inserted in frame between the promoter and *dhfr-ts* ORF as described previously [36]. The *pvdhfr* cassette was then cloned into the pXL-BacII vector [42] at the *Hind*III sites between ITR2 and 3′*histidine-rich protein-2* (Figure 4).

The pHTH and pDCTH transposases were constructed as previously described in [41], [42], respectively. The plasmids for the parasites with transient expression of the *pvdhfr* were developed and transfected as described previously [35].

### Transfections

Transfection of *P. falciparum* NF54 was achieved by parasite invasion of plasmid DNA-loaded RBCs as described earlier [41], [42] with minor modifications. Briefly, mature blood-stage parasites were purified on a MACS magnetic column (Miltentyi Biotec) and 1 million purified parasites were added to erythrocytes loaded with either 300 µg of the transposon plasmid containing either wild-type or various mutant *pvdhfr* and 150 µg of the transposase plasmid pHTH [42] or 300 µg of the transposon plasmid and 300 µg of the transposase plasmid pDCTH [41] to start a 5 ml parasite culture. Individual clones were obtained by limiting dilution of parasites post-drug selection and parasite clones were detected as previously described [57].

### DNA and RNA Extractions

Genomic DNA was extracted from parasites using QIAamp DNA Blood Mini Kits (QIAGEN, USA) and used for: Southern blot hybridisation to confirm the *piggyBac* integration in the *P. falciparum* genome [42]; inverse PCR [42], [58] or vectorette PCR reactions to identify the *piggyBac* insertion sites; quantitative real-time PCR to determine copy number of the *pvdhfr* gene [35].

Total RNA was extracted from parasites using Nucleospin RNA II kits (Macherey-Nagel, Germany) with an additional 1 u/µl DNase I (New England Biolabs, USA) treatment at 37°C for ∼3 hrs, followed by a second round of RNA purification using the same kit. Total RNA was used in quantitative real-time reverse transcription PCR (RT-PCR) to determine the transcription of both *pfdhfr* and *pvdhfr* genes [59].

### Determining CN of pfdhfr and pvdhfr

The CN of the *pfdhfr* and *pvdhfr* was determined by a quantitative real-time PCR in an Mx4000 multiplex quantitative PCR system (Stratagene) using a SYBR green-based assay and *pfdhfr* and *pvdhfr* primers. The single copy gene *eba175* gene was used as a reference (normaliser) gene for estimating the CN for *pfdhfr* and *pvdhfr,* respectively (the target). Primers used to amplify fragments of *pvdhfr* and the *eba175* genes were published previously [35]. The CNs of the plasmids were determined based on the threshold cycle (C*_T_*) values of *pvdhfr* using the ΔΔC*_T_* method [59]. The difference in the CN between the different time points was determined and tested (P value) by using a nonparametric comparison (Spearman-rank test).

### Identification of piggyBac Insertion Sites

*piggyBac* insertion sites in transfected parasites were identified either by inverse PCR [42], [58] or vectorette PCR reactions [60](see Supporting Information S1). The insertion sites were identified as described previously [41].

### Sequencing of pfdhfr

To exclude the possibility that altered drug phenotypes could be due to the mutations or an increase in the copy number of the endogenous *pfdhfr* gene, the *pfdhfr* gene was amplified and sequenced using the primers and PCR conditions described previously [61].

### Analysis of pvdhfr and pfdhfr Transcription

To determine the transcription levels of endogenous *pfdhfr* and transfected *pvdhfr*, quantitative real-time reverse transcription PCR (RT-PCR) assays were performed as described previously [59], using primers specific for *pvdhfr* as described previously [35] and for *pfdhfr* (PfDHFR Fwd 5′ACCTGCGCAGTTCATACACG3’ and PfDHFR Rev 5′TCTTGGGAATGGATAGGGTATTCTGT3’). The statistical significance of the mRNA expression between the transfected parasite lines and their parental strain was determined by either the paired t-test or one way analysis of variance (ANOVA) provided by Sigma Plot for Windows, version 11 (Systat Software, Inc., San Jose, CA).

### In vitro Susceptibility, Bioassay and Time Course Studies

WR99210 and JPC-2067 were kindly supplied by Jacobus Pharmaceutical Company (Princeton, N.J.), Cycloguanil and clociguanil, was purchased from ICI Pharmaceuticals (Macclesfield, United Kingdom) and pyrimethamine, was purchased from Hoffmann-La Roche (Switzerland). All compounds were initially dissolved in dimethyl-sulfoxide (DMSO) and diluted in plasma free LPLF 1640 RPMI. The susceptibility assays were performed as described previously [35], [62].

The bioassays were performed as described previously [49] with minor modifications. Briefly, JPC-2067 working drug solutions (50 µl) were serially diluted by 2-fold on 96 well microtitre plates using complete LPLF 1640 RPMI followed by the addition of 50 µl of infected red blood cells suspended in culture medium. The final cell suspension (100 µl) had a haematocrit of 2%, of which 0.5% were infected erythrocytes (>95% rings).

A solution of 10 µl of 1∶100 dilution of 14.0 Ci/mmol [^3^H] hypoxanthine (Perkin-Elmer): PRPMI (final concentration of 1 µCi/well) was added after 48 hrs when parasites had already reinvaded the red blood cells and were at ring stages, and the parasites were harvested after 96 hrs. Incorporation of ^3^H by intra-erythrocytic malaria parasites (measured in cpm) was recorded for each well, and the concentrations of drugs that inhibited 50% of parasite growth (IC_50_s) when compared with drug-free serum samples (controls) were determined [49]. That is, reduction  = 100 x [(mean counts per minute no drug-control samples – mean counts per minute test samples)/mean counts per minute no-drug control samples]. The percentage of growth inhibition was plotted for each drug concentration. The IC_50_ was determined using nonlinear regression analysis provided by Sigma Plot for Windows, version 11. All assays were performed in duplicate on at least three different occasions. The time point susceptibility assays were conducted as described previously [35]. However, the assays were only performed once in triplicate at each time point.

Statistical tests were performed to compare resistance profiles (IC_50_) between the transfected parasite lines and between them and their parental strain as well as to TM91c235 using either the paired t-test or one way analysis of variance (ANOVA) provided by Sigma Plot for Windows, version 11 (Systat Software, Inc., San Jose, CA).

## Supporting Information

Supporting Information S1 Materials and Methods for inverse PCR or vectorette PCR reactions to identify the *piggyBac* insertion sites.
(DOC)
