# Supplementary material for: Functional Analysis of Plasmodium vivax Dihydrofolate Reductase-Thymidylate Synthase Genes through Stable Transformation of Plasmodium falciparum
Source: PLoS One. 2012 Jul 9;7(7):e40416. doi: 10.1371/journal.pone.0040416 (PMC3392216; doi:10.1371/journal.pone.0040416)
Supplement: Supporting Information S1 — Materials and Methods for inverse PCR or vectorette PCR reactions to identify the piggyBac insertion sites. (DOC) [file pone.0040416.s001.doc]

**Supporting Information S1**

**Materials and Methods**

*Identification of piggyBac insertion sites*

*piggyBac* insertion sites in transformed parasites were identified either by inverse PCR [42,58] or vectorette PCR reactions [60].

*Inverse PCR*

The TAIL PCR was performed as described in [58] with slight modification. Specific primer sets complementary to the sequences flanking the cloning site of the transposon vector were previously described [42]. In addition, four AD primers were used in combination with the specific primers for TAIL-PCR as previously described [58]. Three consecutive PCR reactions were performed. The PCR master mix for the primary PCR reaction consisted of 12.5 µl of GoTaq 2x Master Mix (Promega Inc.), 2 µl of parasitized erythrocytes obtained from the culture pellet as template, 0.5 µl of 10 µM specific primer P1, 5 µl of each 10 µM AD primer and 5 µl of dH_2_O; up to a final volume of 25 µl. The primary PCR conditions were 1 cycle of 94⁰C; followed by 5 cycles of 94⁰C for 30 s, 65⁰C for 1 min, 72⁰C for 2 min; followed by 1 cycle of 94⁰C for 30 s, 25⁰C for 2 min, ramping to 72⁰C over 2 min, 72⁰C for 2 min; followed by 15 cycles of 94⁰C for 30 s, 65⁰C for 1 min, 72⁰C for 2 min, 94⁰C for 30 s, 65⁰C for 1 min, 72⁰C for 2 min, 94⁰C for 30 s, 44⁰C for 1 min, 72⁰C for 2 min; followed by 1 cycle of 72⁰C for 5 min. The PCR master mix for the secondary PCR reaction was the same as the primary PCR reaction master mix except 0.5 µl of 10 µM specific primer P2 replaced P1 specific primer and the template was 2 µl of a 1/40 dilution of the product of the primary reaction. The secondary PCR conditions were 15 cycles of 94⁰C for 30 s, 65⁰C for 1 min, 72⁰C for 2 min, 94⁰C for 30 s, 65⁰C for 1 min, 72⁰C for 2 min, 94⁰C for 30 s, 45⁰C for 1 min and 72⁰C for 2 min followed by 1 cycle of 72⁰C for 5 min. The PCR master mix for the tertiary PCR reaction was the same as the secondary PCR reaction except 0.5 µl of 10 µM specific primer P3 replaced P2 specific primer and the template was 2 µl of a 1/10 dilution of the product of the secondary reaction. The tertiary PCR conditions were 40 cycles of 94⁰C for 30 s, 65⁰C for 1 min and 72⁰C for 2 min followed by 1 cycle of 72⁰C for 5 min.

*Vectorette PCR (vPCR)*

1 µg genomic DNA was extracted from transformed parasites and digested with 10 units of DraI (NEB company) for 1 hour at 37⁰C. The digested DNA was then ligated with 5 µM vectorette, 1 µl of 100uM ATP (Roche), 1 µl of T4 DNA ligase (1 unit/µl; NEB Company) 5 µl of 10x T4 DNA ligase buffer (NEB Company) for 2 cycles of 14-16⁰C for 60 min and 30 min at 37⁰C followed by 1 cycle of 16⁰C for 60 min. The vectorette was created by resuspending each individual vectorette strands (top strand: /5Phos/AAGGAGAGGACGCTGTCTGTCGAAGGTAAGGAACGGACGAGAGAAGGGAGAG3’) and (bottom strand: 5’CTCTCCCTTCTCGAATCGTAACCGTTCGTACGAGAATCGCTGTCCTCTCCTT3’) to 1 mM in TSE buffer, then mixing 5 µl of each vectorette strand together and heating to 95⁰C for 5 min, cooling to room temperature for 3 min and then diluting to 5 µM in TE buffer (Promega). The DNA fragments were amplified via a nested PCR by using 1 µl of the ligation reaction as the template 0.1 µM of universal vectorette primer (5’CGAATCGTAACCGTTCGTACGAGAATCGCT3’) 1 µM of primer P1 for the first reaction and 0.4 µl of the product of the first reaction as the template for the second reaction along with 0.4 µM of nested vectorette primer (5’GTTCGTACGAGAATCGCTGTCCTCTC3’) and 0.4 µM of P2 primer. The PCR conditions for the nested PCR was 7 cycles of 94⁰C for 10 s and 63.5⁰C for 1 min declining by 0.5⁰C for each cycle followed by 32 cycles of 94⁰C for 10 s and 60⁰C for 1 min and 1 cycle of 65⁰C for 7 min.

The amplified PCR products from the tertiary inverse PCR reaction and/or vPCR were sequenced with the P3 primer and analysed using Sequence scanner software V1 (Applied Biosystems) [41].
